# 3-(4-Bromo­phen­yl)-1-phenyl-1*H*-pyrazole-4-carbaldehyde

**DOI:** 10.1107/S1600536811036841

**Published:** 2011-09-17

**Authors:** R. Prasath, P. Bhavana, Seik Weng Ng, Edward R. T. Tiekink

**Affiliations:** a Department of Chemistry, BITS, Pilani – K. K. Birla Goa Campus, Goa 403 726, India; b Department of Chemistry, University of Malaya, 50603 Kuala Lumpur, Malaysia; c Chemistry Department, Faculty of Science, King Abdulaziz University, PO Box 80203 Jeddah, Saudi Arabia

## Abstract

In the title compound, C_16_H_11_BrN_2_O, the phenyl and chloro­benzene rings are twisted out of the mean plane of the pyrazole ring, forming dihedral angles of 13.70 (10) and 36.48 (10)°, respectively. The carbaldehyde group is also twisted out of the pyrazole plane [the C—C—C—O torsion angle is 7.9 (3)°]. A helical supra­molecular chain along the *b* axis and mediated by C—H⋯O inter­actions is the most prominent feature of the crystal packing.

## Related literature

For background details and biological applications of pyrazoles, see: Kaushik *et al.* (2010 ▶); Ali *et al.* (2007 ▶); Krishnamurthy *et al.* (2004 ▶). For a related structure, see: Asiri *et al.* (2011 ▶).
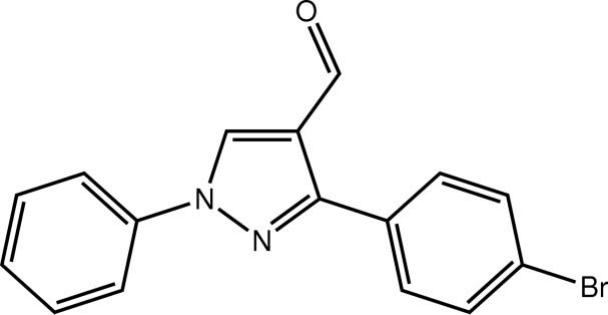


## Experimental

### 

#### Crystal data

C_16_H_11_BrN_2_O
*M* *_r_* = 327.18
Monoclinic, 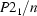

*a* = 17.7233 (4) Å
*b* = 3.8630 (1) Å
*c* = 20.4224 (5) Å
β = 110.137 (3)°
*V* = 1312.75 (6) Å^3^
*Z* = 4
Cu *K*α radiation
μ = 4.23 mm^−1^
*T* = 100 K
0.25 × 0.20 × 0.15 mm

#### Data collection

Agilent SuperNova Dual diffractometer with an Atlas detector
Absorption correction: multi-scan (*CrysAlis PRO*; Agilent, 2010 ▶) *T*_min_ = 0.418, *T*_max_ = 0.569
4619 measured reflections
2593 independent reflections
2542 reflections with *I* > 2σ(*I*)
*R*_int_ = 0.012

#### Refinement

*R*[*F* ^2^ > 2σ(*F* ^2^)] = 0.025
*wR*(*F* ^2^) = 0.069
*S* = 1.02
2593 reflections
181 parameters
H-atom parameters constrained
Δρ_max_ = 0.39 e Å^−3^
Δρ_min_ = −0.66 e Å^−3^

### 

Data collection: *CrysAlis PRO* (Agilent, 2010 ▶); cell refinement: *CrysAlis PRO*; data reduction: *CrysAlis PRO*; program(s) used to solve structure: *SHELXS97* (Sheldrick, 2008 ▶); program(s) used to refine structure: *SHELXL97* (Sheldrick, 2008 ▶); molecular graphics: *ORTEP-3* (Farrugia, 1997 ▶) and *DIAMOND* (Brandenburg, 2006 ▶); software used to prepare material for publication: *publCIF* (Westrip, 2010 ▶).

## Supplementary Material

Crystal structure: contains datablock(s) global, I. DOI: 10.1107/S1600536811036841/hb6403sup1.cif

Structure factors: contains datablock(s) I. DOI: 10.1107/S1600536811036841/hb6403Isup2.hkl

Supplementary material file. DOI: 10.1107/S1600536811036841/hb6403Isup3.cml

Additional supplementary materials:  crystallographic information; 3D view; checkCIF report

## Figures and Tables

**Table 1:** Hydrogen-bond geometry (Å, °)

*D*—H⋯*A*	*D*—H	H⋯*A*	*D*⋯*A*	*D*—H⋯*A*
C12—H12⋯O1^i^	0.95	2.49	3.435 (2)	171
C16—H16⋯O1^ii^	0.95	2.46	3.288 (3)	145

Symmetry codes: (i) 
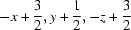
; (ii) 
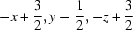
.

**Fig. 1.**
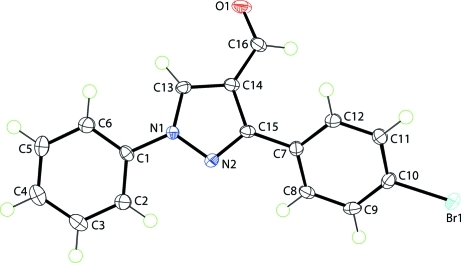
The molecular structures of (I) showing displacement ellipsoids at the 70% probability level.

**Fig. 2.**
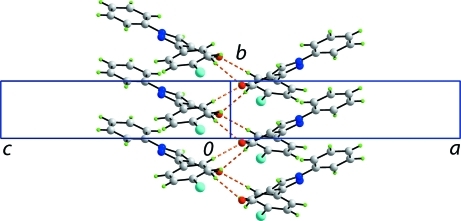
Helical supramolecular chain in (I) mediated by C—H···O (orange dashed lines) interactions.

## supplementary crystallographic information

### Comment

A broad spectrum of biological activities [anti-bacterial, anti-depressant, anti-convulsive, anti-hypertensive, anti-oxidant anti-viral and anti-tumour] have been noted for pyrazoles and their derivatives (Kaushik *et al.*, 2010; Ali *et al.*, 2007; Krishnamurthy *et al.*, 2004). In continuation of structural studies in this area (Asiri *et al.*, 2011), the title compound, (I), was investigated.

The dihedral angles formed between the central pyrazole ring [r.m.s. deviation = 0.003 Å] and the N– and C-bound benzene rings of 13.70 (10) and 36.48 (10) °, respectively, indicate significant twists in the molecule of (I), Fig. 1. Similarly, the carbaldehyde group is twisted out of the plane of the five-membered ring as seen in the value of the C13—C14—C16—O1 torsion angle of 7.9 (3) °. The relative disposition of the benzene rings preclude close intermolecular association with the imine-N2 atom which, indeed, forms a close intramolecular C2—H···N2 contact, Table 1.

The crystal packing features C—H···O interactions involving a bifurcated carbonyl-O1 atom, Table 1. These result in the formation of a helical supramolecular chain along the *b* axis, Fig. 2.

### Experimental

Phosphoryl chloride (5.6 ml) was added drop wise to cold *N*,*N*-dimethylformamide (22.5 ml) under continuous stirring at 273–278 K for about 30 min. 4-Bromoacetophenone phenylhydrazone (5 g, 17 mmol) was added to the above reaction mixture. The resulting mixture was further stirred at 333 K for 6 h. and cooled to room temperature. The crude product was poured into crushed ice which resulted in a white precipitate. The resultant solid was filtered, dried and purified by column chromatography using chloroform. Recrystallization was by slow evaporation of chloroform solution of (I) which yielded colourless prisms. *M*.pt. 413–415 K. Yield: 56%.

### Refinement

Carbon-bound H-atoms were placed in calculated positions [C—H 0.95 Å, *U*_iso_(H) = 1.2*U*_eq_(C)] and were included in the refinement in the riding model approximation.

### Crystal data

**Table:**

C_16_H_11_BrN_2_O	*F*(000) = 656
*M**_r_* = 327.18	*D*_x_ = 1.655 Mg m^−^^3^
Monoclinic, *P*2_1_/*n*	Cu *K*α radiation, λ = 1.54184 Å
Hall symbol: -P 2yn	Cell parameters from 3680 reflections
*a* = 17.7233 (4) Å	θ = 2.7–74.1°
*b* = 3.8630 (1) Å	µ = 4.23 mm^−^^1^
*c* = 20.4224 (5) Å	*T* = 100 K
β = 110.137 (3)°	Prism, colourless
*V* = 1312.75 (6) Å^3^	0.25 × 0.20 × 0.15 mm
*Z* = 4	

### Data collection

**Table:**

Agilent SuperNova Dual diffractometer with an Atlas detector	2593 independent reflections
Radiation source: SuperNova (Cu) X-ray Source	2542 reflections with *I* > 2σ(*I*)
Mirror	*R*_int_ = 0.012
Detector resolution: 10.4041 pixels mm^-1^	θ_max_ = 74.3°, θ_min_ = 2.9°
ω scans	*h* = −21→21
Absorption correction: multi-scan (*CrysAlis PRO*; Agilent, 2010)	*k* = −4→4
*T*_min_ = 0.418, *T*_max_ = 0.569	*l* = −19→25
4619 measured reflections	

### Refinement

**Table:**

Refinement on *F*^2^	Primary atom site location: structure-invariant direct methods
Least-squares matrix: full	Secondary atom site location: difference Fourier map
*R*[*F*^2^ > 2σ(*F*^2^)] = 0.025	Hydrogen site location: inferred from neighbouring sites
*wR*(*F*^2^) = 0.069	H-atom parameters constrained
*S* = 1.02	*w* = 1/[σ^2^(*F*_o_^2^) + (0.0432*P*)^2^ + 1.2934*P*] where *P* = (*F*_o_^2^ + 2*F*_c_^2^)/3
2593 reflections	(Δ/σ)_max_ = 0.004
181 parameters	Δρ_max_ = 0.39 e Å^−^^3^
0 restraints	Δρ_min_ = −0.66 e Å^−^^3^

### Special details

**Table:**

Geometry. All s.u.'s (except the s.u. in the dihedral angle between two l.s. planes) are estimated using the full covariance matrix. The cell s.u.'s are taken into account individually in the estimation of s.u.'s in distances, angles and torsion angles; correlations between s.u.'s in cell parameters are only used when they are defined by crystal symmetry. An approximate (isotropic) treatment of cell s.u.'s is used for estimating s.u.'s involving l.s. planes.
Refinement. Refinement of *F*^2^ against ALL reflections. The weighted *R*-factor *wR* and goodness of fit *S* are based on *F*^2^, conventional *R*-factors *R* are based on *F*, with *F* set to zero for negative *F*^2^. The threshold expression of *F*^2^ > 2σ(*F*^2^) is used only for calculating *R*-factors(gt) *etc*. and is not relevant to the choice of reflections for refinement. *R*-factors based on *F*^2^ are statistically about twice as large as those based on *F*, and *R*- factors based on ALL data will be even larger.

### Fractional atomic coordinates and isotropic or equivalent isotropic displacement parameters (Å^2^)

**Table:**

	*x*	*y*	*z*	*U*_iso_*/*U*_eq_	
Br1	0.979494 (11)	0.14952 (5)	1.111751 (9)	0.01554 (9)	
O1	0.66107 (9)	0.4209 (4)	0.71084 (7)	0.0183 (3)	
N1	0.54596 (9)	0.8378 (4)	0.84419 (8)	0.0106 (3)	
N2	0.60504 (9)	0.7642 (4)	0.90629 (8)	0.0114 (3)	
C1	0.47250 (10)	0.9916 (5)	0.84392 (9)	0.0112 (3)	
C2	0.45581 (11)	0.9963 (5)	0.90561 (10)	0.0146 (4)	
H2	0.4929	0.9008	0.9471	0.018*	
C3	0.38437 (12)	1.1422 (5)	0.90583 (11)	0.0170 (4)	
H3	0.3729	1.1496	0.9480	0.020*	
C4	0.32952 (12)	1.2773 (5)	0.84520 (11)	0.0169 (4)	
H4	0.2805	1.3744	0.8456	0.020*	
C5	0.34673 (11)	1.2698 (5)	0.78370 (10)	0.0165 (4)	
H5	0.3092	1.3617	0.7421	0.020*	
C6	0.41865 (12)	1.1284 (5)	0.78272 (10)	0.0148 (4)	
H6	0.4307	1.1255	0.7408	0.018*	
C7	0.73867 (11)	0.5078 (5)	0.94282 (9)	0.0112 (4)	
C8	0.73831 (12)	0.3647 (5)	1.00557 (10)	0.0128 (4)	
H8	0.6888	0.3396	1.0135	0.015*	
C9	0.80911 (12)	0.2589 (5)	1.05641 (9)	0.0141 (4)	
H9	0.8085	0.1639	1.0991	0.017*	
C10	0.88090 (11)	0.2942 (5)	1.04391 (10)	0.0135 (4)	
C11	0.88296 (11)	0.4357 (5)	0.98216 (10)	0.0146 (4)	
H11	0.9326	0.4580	0.9744	0.018*	
C12	0.81210 (11)	0.5442 (5)	0.93190 (9)	0.0133 (4)	
H12	0.8133	0.6440	0.8898	0.016*	
C13	0.56590 (11)	0.7418 (5)	0.78887 (9)	0.0117 (3)	
H13	0.5340	0.7694	0.7411	0.014*	
C14	0.64162 (11)	0.5954 (5)	0.81478 (10)	0.0115 (4)	
C15	0.66323 (11)	0.6177 (5)	0.88888 (9)	0.0105 (4)	
C16	0.68436 (11)	0.4243 (5)	0.77447 (10)	0.0135 (4)	
H16	0.7332	0.3085	0.7991	0.016*	

### Atomic displacement parameters (Å^2^)

**Table:**

	*U*^11^	*U*^22^	*U*^33^	*U*^12^	*U*^13^	*U*^23^
Br1	0.01392 (13)	0.01863 (13)	0.01135 (12)	0.00306 (7)	0.00088 (9)	0.00292 (7)
O1	0.0176 (7)	0.0277 (7)	0.0116 (6)	−0.0046 (6)	0.0076 (5)	−0.0054 (6)
N1	0.0085 (7)	0.0138 (8)	0.0094 (7)	0.0002 (6)	0.0029 (6)	−0.0001 (5)
N2	0.0103 (7)	0.0144 (7)	0.0089 (7)	0.0004 (6)	0.0024 (6)	0.0000 (6)
C1	0.0089 (8)	0.0110 (9)	0.0143 (8)	−0.0015 (7)	0.0048 (7)	−0.0025 (7)
C2	0.0142 (9)	0.0164 (9)	0.0134 (8)	0.0000 (7)	0.0051 (7)	−0.0003 (7)
C3	0.0168 (10)	0.0180 (10)	0.0195 (10)	−0.0001 (7)	0.0103 (8)	−0.0031 (7)
C4	0.0130 (9)	0.0141 (9)	0.0246 (10)	−0.0002 (8)	0.0076 (8)	−0.0026 (8)
C5	0.0113 (9)	0.0161 (9)	0.0192 (9)	0.0009 (8)	0.0014 (7)	0.0006 (8)
C6	0.0139 (9)	0.0172 (10)	0.0136 (9)	−0.0002 (7)	0.0051 (8)	0.0002 (7)
C7	0.0107 (8)	0.0111 (9)	0.0113 (8)	0.0002 (7)	0.0032 (7)	−0.0014 (7)
C8	0.0131 (9)	0.0150 (9)	0.0127 (9)	−0.0004 (7)	0.0075 (7)	−0.0008 (7)
C9	0.0190 (9)	0.0149 (9)	0.0097 (8)	0.0004 (8)	0.0067 (7)	0.0011 (7)
C10	0.0129 (9)	0.0141 (9)	0.0110 (8)	0.0007 (7)	0.0010 (7)	−0.0009 (7)
C11	0.0106 (9)	0.0200 (9)	0.0138 (9)	−0.0019 (7)	0.0049 (7)	0.0012 (8)
C12	0.0135 (9)	0.0167 (9)	0.0108 (8)	−0.0006 (7)	0.0053 (7)	0.0018 (7)
C13	0.0123 (8)	0.0145 (9)	0.0082 (8)	−0.0018 (7)	0.0034 (7)	−0.0014 (7)
C14	0.0119 (8)	0.0132 (8)	0.0103 (8)	−0.0016 (7)	0.0051 (7)	−0.0010 (7)
C15	0.0114 (9)	0.0117 (8)	0.0094 (9)	−0.0026 (6)	0.0048 (7)	−0.0009 (6)
C16	0.0118 (8)	0.0168 (9)	0.0134 (9)	−0.0020 (7)	0.0064 (7)	−0.0031 (7)

### Geometric parameters (Å, °)

**Table:**

Br1—C10	1.9023 (19)	C7—C8	1.398 (3)
O1—C16	1.220 (2)	C7—C12	1.401 (2)
N1—C13	1.347 (2)	C7—C15	1.472 (3)
N1—N2	1.368 (2)	C8—C9	1.387 (3)
N1—C1	1.429 (2)	C8—H8	0.9500
N2—C15	1.328 (2)	C9—C10	1.388 (3)
C1—C6	1.390 (3)	C9—H9	0.9500
C1—C2	1.390 (3)	C10—C11	1.386 (3)
C2—C3	1.387 (3)	C11—C12	1.385 (3)
C2—H2	0.9500	C11—H11	0.9500
C3—C4	1.386 (3)	C12—H12	0.9500
C3—H3	0.9500	C13—C14	1.383 (3)
C4—C5	1.391 (3)	C13—H13	0.9500
C4—H4	0.9500	C14—C15	1.430 (2)
C5—C6	1.393 (3)	C14—C16	1.455 (3)
C5—H5	0.9500	C16—H16	0.9500
C6—H6	0.9500		
			
C13—N1—N2	112.47 (15)	C9—C8—H8	119.5
C13—N1—C1	127.81 (16)	C7—C8—H8	119.5
N2—N1—C1	119.70 (15)	C8—C9—C10	118.95 (17)
C15—N2—N1	104.93 (14)	C8—C9—H9	120.5
C6—C1—C2	121.05 (17)	C10—C9—H9	120.5
C6—C1—N1	120.28 (16)	C11—C10—C9	121.26 (17)
C2—C1—N1	118.67 (17)	C11—C10—Br1	118.24 (14)
C3—C2—C1	119.17 (18)	C9—C10—Br1	120.50 (14)
C3—C2—H2	120.4	C12—C11—C10	119.49 (17)
C1—C2—H2	120.4	C12—C11—H11	120.3
C2—C3—C4	120.68 (18)	C10—C11—H11	120.3
C2—C3—H3	119.7	C11—C12—C7	120.44 (17)
C4—C3—H3	119.7	C11—C12—H12	119.8
C3—C4—C5	119.61 (18)	C7—C12—H12	119.8
C3—C4—H4	120.2	N1—C13—C14	106.98 (16)
C5—C4—H4	120.2	N1—C13—H13	126.5
C4—C5—C6	120.48 (19)	C14—C13—H13	126.5
C4—C5—H5	119.8	C13—C14—C15	104.56 (16)
C6—C5—H5	119.8	C13—C14—C16	126.51 (17)
C1—C6—C5	118.99 (18)	C15—C14—C16	128.61 (17)
C1—C6—H6	120.5	N2—C15—C14	111.05 (16)
C5—C6—H6	120.5	N2—C15—C7	120.77 (16)
C8—C7—C12	118.90 (17)	C14—C15—C7	128.17 (16)
C8—C7—C15	120.71 (16)	O1—C16—C14	123.78 (18)
C12—C7—C15	120.40 (16)	O1—C16—H16	118.1
C9—C8—C7	120.97 (17)	C14—C16—H16	118.1
			
C13—N1—N2—C15	−0.1 (2)	Br1—C10—C11—C12	−179.81 (15)
C1—N1—N2—C15	178.39 (16)	C10—C11—C12—C7	−0.8 (3)
C13—N1—C1—C6	−14.2 (3)	C8—C7—C12—C11	0.9 (3)
N2—N1—C1—C6	167.59 (17)	C15—C7—C12—C11	−178.95 (18)
C13—N1—C1—C2	164.85 (18)	N2—N1—C13—C14	0.3 (2)
N2—N1—C1—C2	−13.4 (3)	C1—N1—C13—C14	−178.04 (17)
C6—C1—C2—C3	−0.4 (3)	N1—C13—C14—C15	−0.3 (2)
N1—C1—C2—C3	−179.44 (17)	N1—C13—C14—C16	173.58 (18)
C1—C2—C3—C4	1.0 (3)	N1—N2—C15—C14	−0.1 (2)
C2—C3—C4—C5	−0.7 (3)	N1—N2—C15—C7	178.76 (16)
C3—C4—C5—C6	−0.1 (3)	C13—C14—C15—N2	0.3 (2)
C2—C1—C6—C5	−0.4 (3)	C16—C14—C15—N2	−173.44 (19)
N1—C1—C6—C5	178.59 (18)	C13—C14—C15—C7	−178.49 (18)
C4—C5—C6—C1	0.7 (3)	C16—C14—C15—C7	7.8 (3)
C12—C7—C8—C9	−0.2 (3)	C8—C7—C15—N2	37.3 (3)
C15—C7—C8—C9	179.64 (17)	C12—C7—C15—N2	−142.86 (19)
C7—C8—C9—C10	−0.6 (3)	C8—C7—C15—C14	−143.99 (19)
C8—C9—C10—C11	0.7 (3)	C12—C7—C15—C14	35.8 (3)
C8—C9—C10—Br1	−179.51 (15)	C13—C14—C16—O1	7.9 (3)
C9—C10—C11—C12	0.0 (3)	C15—C14—C16—O1	−179.63 (19)

### Hydrogen-bond geometry (Å, °)

**Table:**

*D*—H···*A*	*D*—H	H···*A*	*D*···*A*	*D*—H···*A*
C12—H12···O1^i^	0.95	2.49	3.435 (2)	171
C16—H16···O1^ii^	0.95	2.46	3.288 (3)	145

Symmetry codes: (i) −*x*+3/2, *y*+1/2, −*z*+3/2; (ii) −*x*+3/2, *y*−1/2, −*z*+3/2.

## References

[bb1] Agilent (2010). *CrysAlis PRO* Agilent Technologies, Yarnton, England.

[bb2] Ali, M. A., Shaharyar, M., Siddiqui, A. A., Sriram, D., Yogeeswari, P. & Clercq, E. D. (2007). *Acta Pol. Pharm.* **63**, 423–428. 18540162

[bb3] Asiri, A. M., Al-Youbi, A. O., Alamry, K. A., Faidallah, H. M., Ng, S. W. & Tiekink, E. R. T. (2011). *Acta Cryst.* E**67**, o2157. 10.1107/S1600536811029473 PMC3213593 22091170

[bb4] Brandenburg, K. (2006). *DIAMOND* Crystal Impact GbR, Bonn, Germany.

[bb5] Farrugia, L. J. (1997). *J. Appl. Cryst.* **30**, 565.

[bb6] Kaushik, D., Khan, S. A., Chawla, G. & Kumar, S. (2010). *Eur. J. Med. Chem.* **45**, 3943–3949. 10.1016/j.ejmech.2010.05.049 20573423

[bb7] Krishnamurthy, M., Li, W. & Moore, B. M. (2004). *Bioorg. Med. Chem.* **12**, 393–404. 10.1016/j.bmc.2003.10.045 14723958

[bb8] Sheldrick, G. M. (2008). *Acta Cryst.* A**64**, 112–122. 10.1107/S0108767307043930 18156677

[bb9] Westrip, S. P. (2010). *J. Appl. Cryst.* **43**, 920–925.

